# Supplementary material for: Highly tunable band structure in ferroelectric R-stacked bilayer WSe2
Source: Nat Commun. 2026 Feb 6;17:2457. doi: 10.1038/s41467-026-68854-x (PMC12992584; doi:10.1038/s41467-026-68854-x)
Supplement: Supplementary file 1 — Supplementary Information [file 41467_2026_68854_MOESM1_ESM.pdf]

Supplementary Information: Highly tunable band structure in ferroelectric R-stacked bilayer WSe_2

Zhe Li,[1] Prokhor Thor,[1] George Kourmoulakis,[1] Tatyana V. Ivanova,[1] Takashi Taniguchi,[2] Kenji Watanabe,[3] Hongyi Yu,[4,5] Mauro Brotons-Gisbert,[1] and Brian D. Gerardot[1,*]

[1]*Institute of Photonics and Quantum Sciences, SUPA, Heriot-Watt University, Edinburgh EH14 4AS, UK*

[2]*Research Center for Materials Nanoarchitectonics, National Institute for Materials Science, 1-1 Namiki, Tsukuba 305-0044, Japan*

[3]*Research Center for Electronic and Optical Materials, National Institute for Materials Science, 1-1 Namiki, Tsukuba 305-0044, Japan*

[4]*Guangdong Provincial Key Laboratory of Quantum Metrology and Sensing & School of Physics and Astronomy, Sun Yat-Sen University (Zhuhai Campus), Zhuhai 519082, China*

[5]*State Key Laboratory of Optoelectronic Materials and Technologies, Sun Yat-Sen University (Guangzhou Campus), Guangzhou 510275, China*

Supplementary Information: Table of Contents

- Supplementary Methods
  - Reflectance Contrast ($\Delta R/R_0$) Calculation and Normalization
  - Doping Concentration Calculation
  - g-factor Calculation
- Supplementary Figure 1: Optical characterization and schematic of the bilayer WSe_2 device.
- Supplementary Figure 2: Kelvin probe force microscopy (KPFM) map of an unencapsulated R-stacked bilayer WSe_2.
- Supplementary Figure 3: Robust ferroelectric domain formation in an encapsulated control sample.
- Supplementary Figure 4: Optical uniformity and reproducibility of the encapsulated device.
- Supplementary Figure 5: Top-gate / bottom-gate voltage map showing experimental voltage-sweep paths.
- Supplementary Figure 6: Magnetic-field-dependent reflectance contrast under different doping conditions.
- Supplementary Figure 7: Helicity-resolved reflectance contrast spectra and Zeeman splitting fit.
- Supplementary Figure 8: Doping dependence of reflectance contrast in a single-domain region at small displacement fields.
- Supplementary Figure 9: Doping dependence after domain switch by a training field ($0.15\ \mathrm{V\,nm^{-1}}$).
- Supplementary Figure 10: Comparison of domains' responses at a fixed displacement field.

- Supplementary Figure 11: Ferroelectric switching in n-doped R-stacked bilayer WSe_2.
- Supplementary Figure 12: Doping dependence near the valence-band-maximum switching field.
- Supplementary Figure 13: Displacement field dependent interlayer exciton emission.

SUPPLEMENTARY METHODS

Reflectance Contrast Measurement and Normalization

We focus a broadband white-light source from a power-stabilized halogen lamp onto the sample, and measure the reflected signal with a spectrometer equipped with a liquid-nitrogen-cooled charge-coupled device (CCD). To obtain clear excitonic resonances, we calculate the differential reflectance contrast $\Delta R/R_0$ using the formula:

$$\frac{\Delta R}{R_0} = \frac{R_{\text{sample}} - R_0}{R_0}$$

where R_{sample} is the raw reflectance spectrum collected from the dual-gated bilayer WSe_2 region, and R_0 is the background reflectance spectrum collected from an adjacent heterostructure area that contains the hBN and graphene layers but not the WSe_2 bilayer. This calculation effectively removes the spectral response of the substrate, optical elements, and detector.

In Figure 3c of the main text, a normalization procedure was applied to compare the relative contributions of coexisting ferroelectric domains. For this, we integrate the X_H^0 intensity in an energetic window around its resonance. Since the X_H^0 excitonic resonance corresponds to a negative dip in the reflectance spectra, we invert the integrated area to correlate it with the oscillator strength. This integrated oscillator strength is then normalized by the maximum value observed across the doping range. This procedure allows for a direct comparison of how the oscillator strength is distributed between the coexisting AB and BA domains. Critically, the asymmetric lineshape of the AP_H^+ resonance can contribute a non-linear background, which is reflected in the baseline observed in Figure 3c even when the X_H^0 oscillator strength disappears.

Doping Concentration Calculation

The doping concentration (carrier density) n induced by the dual-gate structure is evaluated using a parallel-plate capacitor model. The top gate (V_{tg}) and bottom gate (V_{bg}) induce charge densities n_{tg} and n_{bg}, respectively:

$$n_{tg} = \frac{C_{tg} V_{tg}}{e}$$

$$n_{bg} = \frac{C_{bg}V_{bg}}{e}$$

where $C_{tg} = \varepsilon_0\varepsilon_{\text{hBN}}/d_t$ and $C_{bg} = \varepsilon_0\varepsilon_{\text{hBN}}/d_b$ are the geometric capacitances per unit area of the top and bottom dielectric layers. Here, e is the elementary charge, $\varepsilon_0 = 8.854 \times 10^{-12}$ F/m is the vacuum permittivity, and $\varepsilon_{\text{hBN}} \approx 3.8$ is the relative permittivity of the hBN dielectric. d_t and d_b are the thicknesses of the top and bottom dielectrics. The total induced carrier density in the WSe_2 bilayer is the sum of the contributions from both gates:

$$n = n_{tg} + n_{bg}$$

g-factor Calculation

The exciton g-factors were extracted from helicity-resolved reflectance contrast measurements under an out-of-plane magnetic field B. We apply a magnetic field from $-5\,\text{T}$ to $5\,\text{T}$ and excite the sample with linearly polarized light. The reflected signal is then resolved into right-handed (σ^+) and left-handed (σ^-) circular polarization bases to observe the Zeeman splitting of the excitonic complexes in the $\pm$K valleys.

The Zeeman splitting is defined as $\Delta E = E_{\sigma+} - E_{\sigma-}$, where $E_{\sigma+}$ and $E_{\sigma-}$ are the exciton peak energies for each polarization. As shown in Figure S6a-b, the peak energies for X^0_L and X^0_H at each magnetic field are determined by fitting the spectral peaks with a double Lorentzian function. This splitting is linearly proportional to the applied magnetic field:

$$\Delta E = g\mu_B B$$

where g is the exciton g-factor and μ_B is the Bohr magneton. The g-factor is then extracted by performing a linear fit of the experimentally measured Zeeman splitting ΔE as a function of B, as shown in Figure S6c-d. The slope of this line is equal to $g\mu_B$.

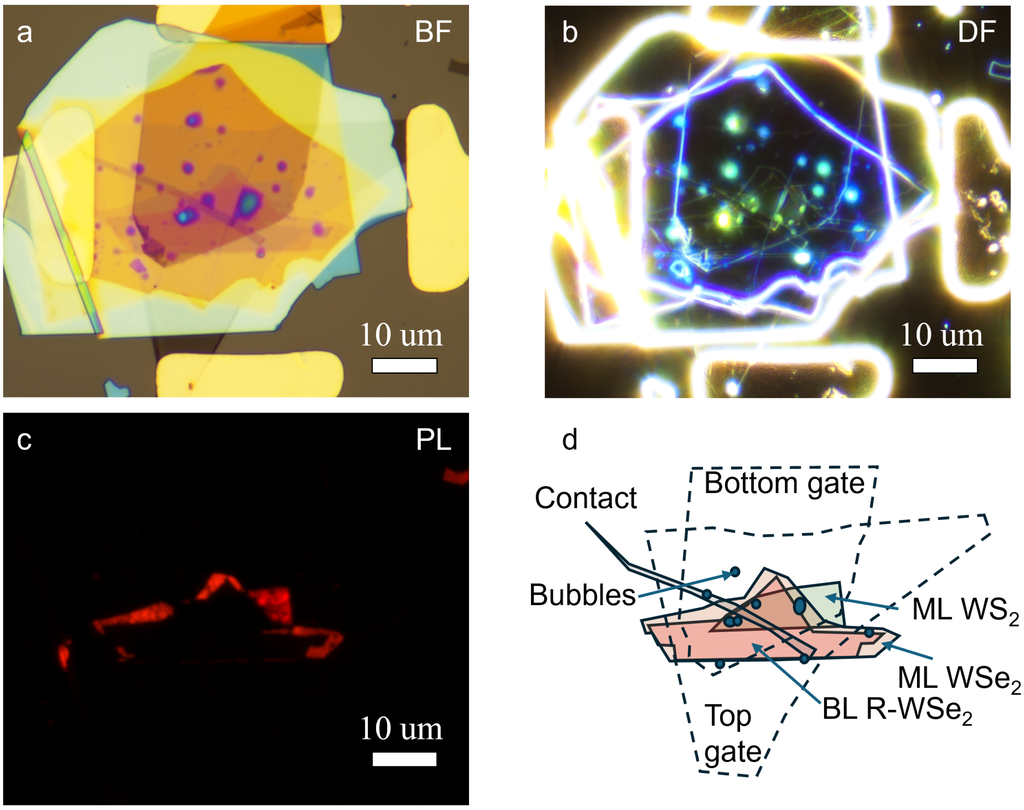

Supplementary Figure 1: Optical characterization and schematic of the bilayer WSe_2 device. (**a**) Bright-field (BF) and (**b**) Dark-field (DF) optical images of the device. The boundaries of the monolayer (ML) and bilayer (BL) WSe_2 regions are clearly visible. (**c**) Photoluminescence (PL) intensity image of the sample. The ML region shows strong, uniform PL, whereas the BL region exhibits strong and highly uniform PL quenching, indicating robust and homogeneous interlayer coupling. (**d**) Device schematic illustrating the ML and BL areas, the top and bottom graphite gates, the graphite contact, and bubbles. The experimental data were collected from the dual-gated BL region, which these optical data collectively demonstrate is highly uniform and possesses a high-quality interface. The experimental data, measured on the dual-gated BL region, collectively demonstrate that it is highly uniform and possesses a high-quality interface.

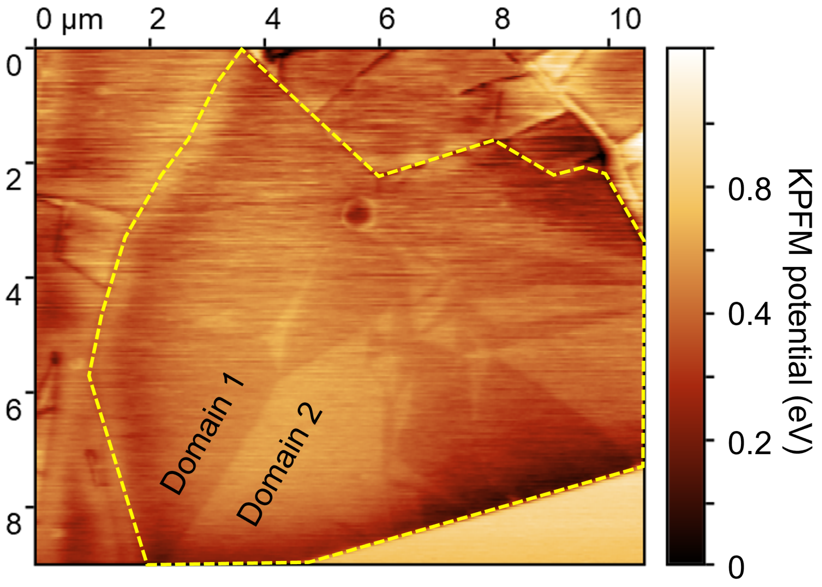

Supplementary Figure 2: Kelvin probe force microscopy (KPFM) map of an unencapsulated R-stacked bilayer WSe_2. The yellow dashed outline marks the bilayer WSe_2 region. Two distinct KPFM potentials are observed within the outlined region, which we attribute to domains with different stacking orders (AB vs BA). Domains vary in size; large domains can extend over several micrometers. The observed potential contrast is consistent with stacking-order–dependent surface potential and supports the presence of ferroelectric domains in R-stacked bilayer WSe_2. This measurement was performed on a separate sample from the main encapsulated (gated) device used for optical measurements, as KPFM requires an exposed surface. The purpose of this figure is to provide independent validation of intrinsic ferroelectric domain formation. We note that the absolute potential values are subject to a large, systematic offset inherent to the KPFM measurement, resulting in a positive shift for both domains. The key observation is the distinct relative potential difference between the domains, which confirms their opposite built-in polarization.

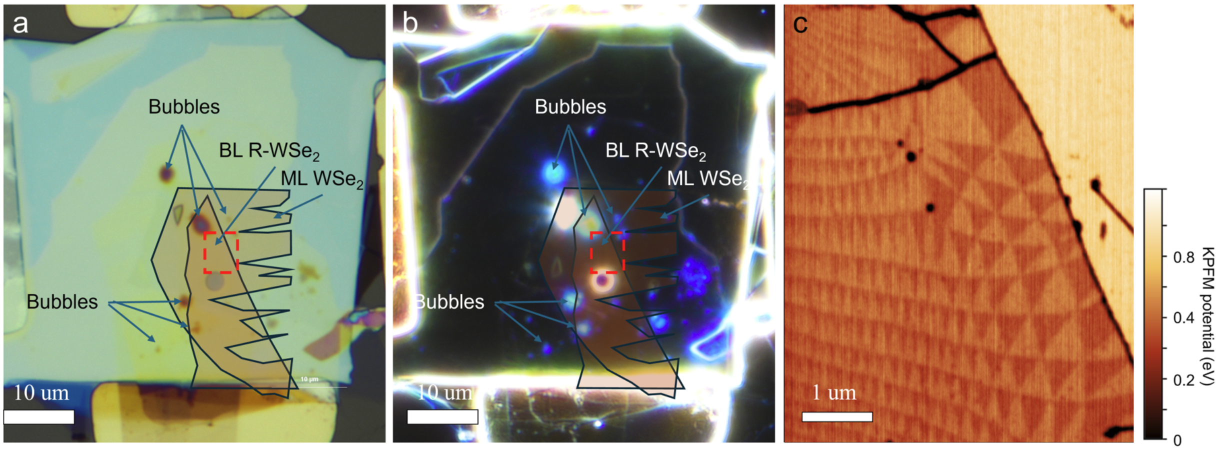

Supplementary Figure 3: Robust ferroelectric domain formation in an encapsulated control sample. To further verify that ferroelectric domains form reliably despite the presence of interface bubbles, we fabricated a control heterostructure (2 nm top hBN / R-stacked bilayer WSe_2 / graphite contact / bottom hBN / bottom Graphite) utilizing the same tear-and-stack technique. (a) Bright-field and (b) dark-field optical images of the control sample. Similar to the device used in the main text, bubbles are visible across the encapsulation area (indicated by arrows). (c) KPFM surface potential map corresponding to the region marked by red dashed boxes in (a) and (b). Despite the presence of optically visible bubbles, the KPFM scan reveals clear, well-defined ferroelectric domains with distinct potential contrast. This confirms that the bubbles generally reside at non-critical interfaces (e.g., hBN/graphite) and do not disrupt the formation of the ferroelectric moiré interface or the resulting polarization domains.

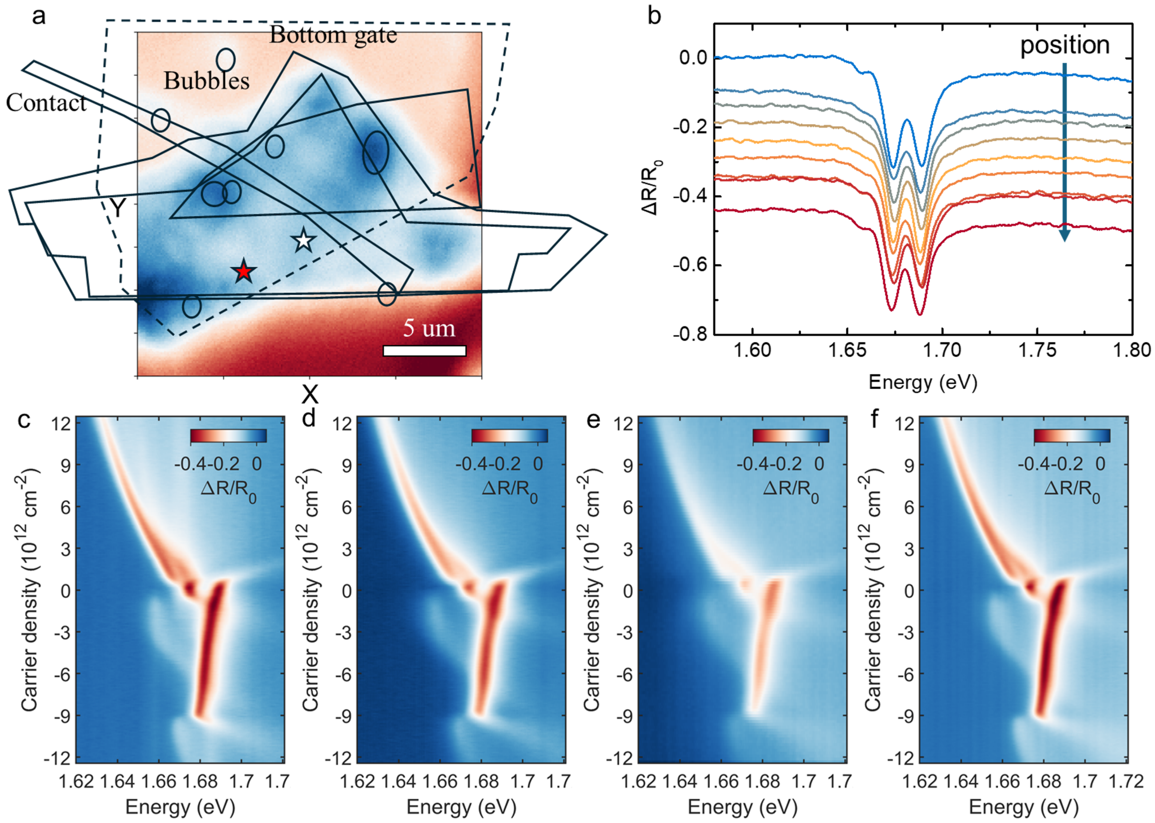

Supplementary Figure 4: Optical uniformity and reproducibility of the encapsulated device. (**a**) 4K reflectance map of the device, generated by integrating the spectral intensity between 1.65 eV and 1.70 eV. A schematic of the device stack is overlaid for clarity. The reflectance signal is influenced by both the WSe_2 bilayer (BL) and the graphite gates. The map demonstrates the high spatial uniformity of the BL WSe_2 area. The red and white stars indicate the measurement locations for the data presented in the main manuscript, corresponding to a mixed-domain and a large single-domain region, respectively. (**b**) Reflectance contrast spectra from nine locations along the red and white star direction. Despite minor variations in peak characteristics (position, intensity, and width), all spectra clearly resolve the distinct LE and HE exciton peaks. (**c-f**) Doping-dependent reflectance contrast spectra collected from four additional locations on the device (distinct from those in the main text). While the signal-to-noise ratio varies (e.g., weaker intensity in (e)), the spectral evolution trend with doping is identical across all locations, confirming the reproducibility of the reported exciton-polaron physics.

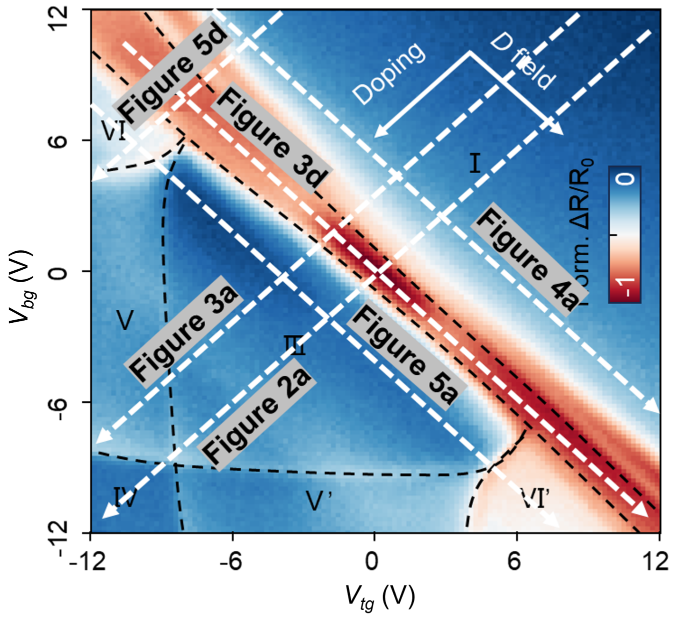

Supplementary Figure 5: Top-gate / bottom-gate voltage map and experimental sweep paths. Two-dimensional map of the applied top-gate and bottom-gate voltages. Dashed arrows indicate the specific gate-voltage sweep paths used for the various measurements in the main text.

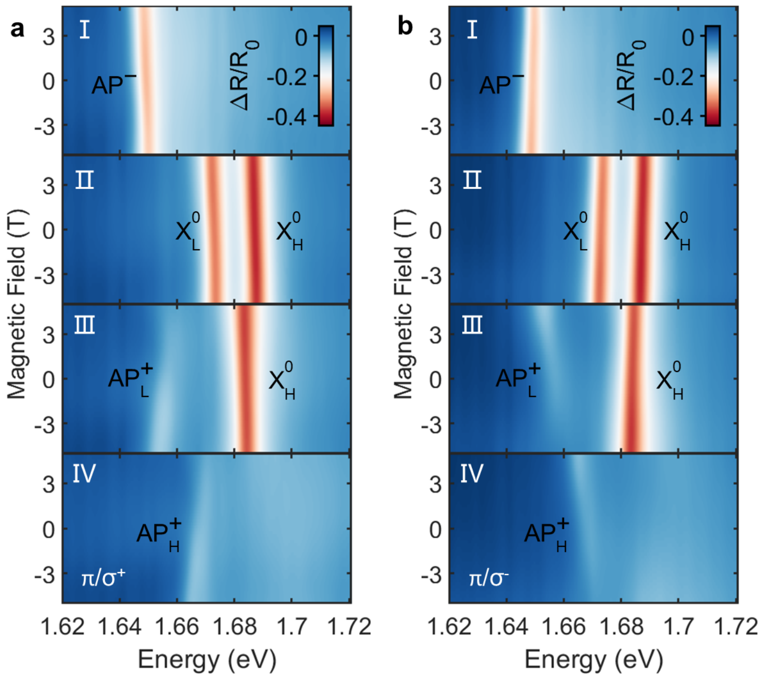

Supplementary Figure 6: Magnetic-field-dependent reflectance contrast under different doping conditions. Reflectance-contrast spectra recorded as a function of magnetic field across several doping points. **a**, Spectra measured with linear polarization excitation and σ^+ detection. **b**, Spectra measured with linear polarization excitation and σ^- detection.

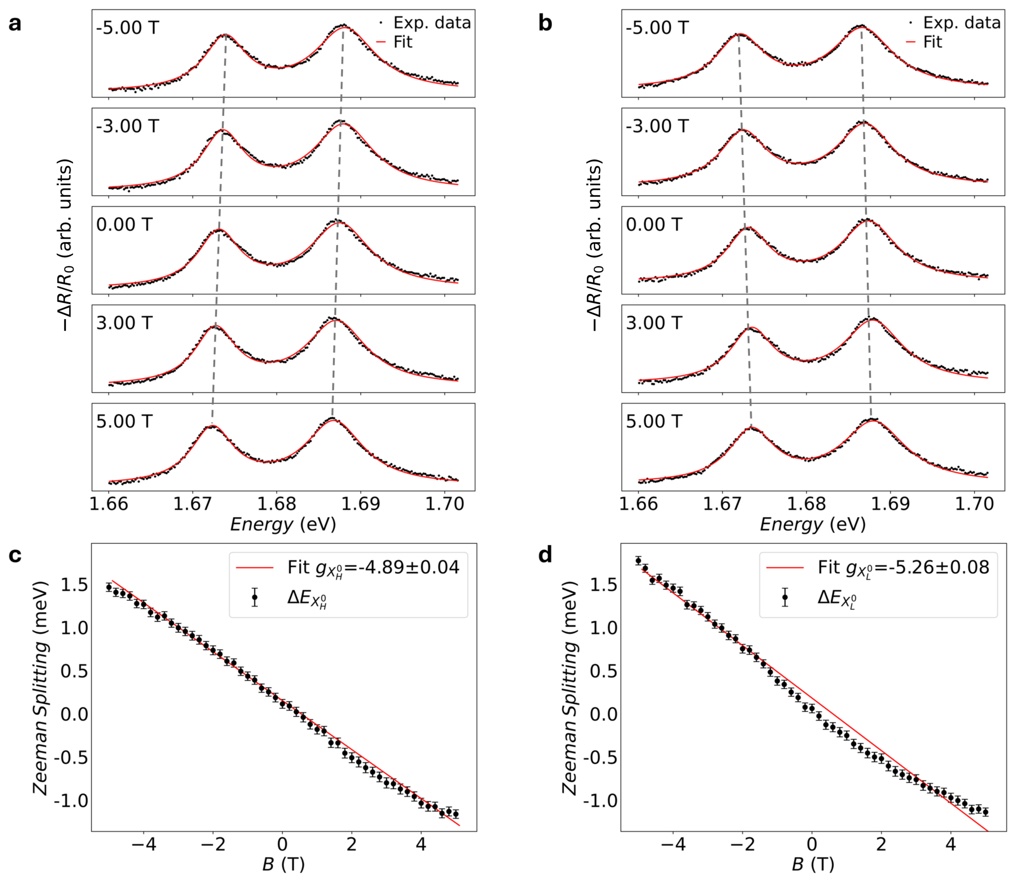

Supplementary Figure 7: Helicity-resolved reflectance contrast spectra and Zeeman splitting fit. We show the reflectance contrast spectra under (**a**) σ^+ and (**b**) σ^- detection under five varying magnetic fields B between -5 T and 5 T. The experimental data was fitted using a double Lorentzian. A grey dashed line acts as a visual guide to show the energetic shift of the X_H^0 and X_L^0 peaks. (**c**) Linear fit (red line) of the Zeeman splitting of X_H^0 and (**d**) X_L^0 to extract the g-factor. Error bars indicate the standard error of the Zeeman splitting.

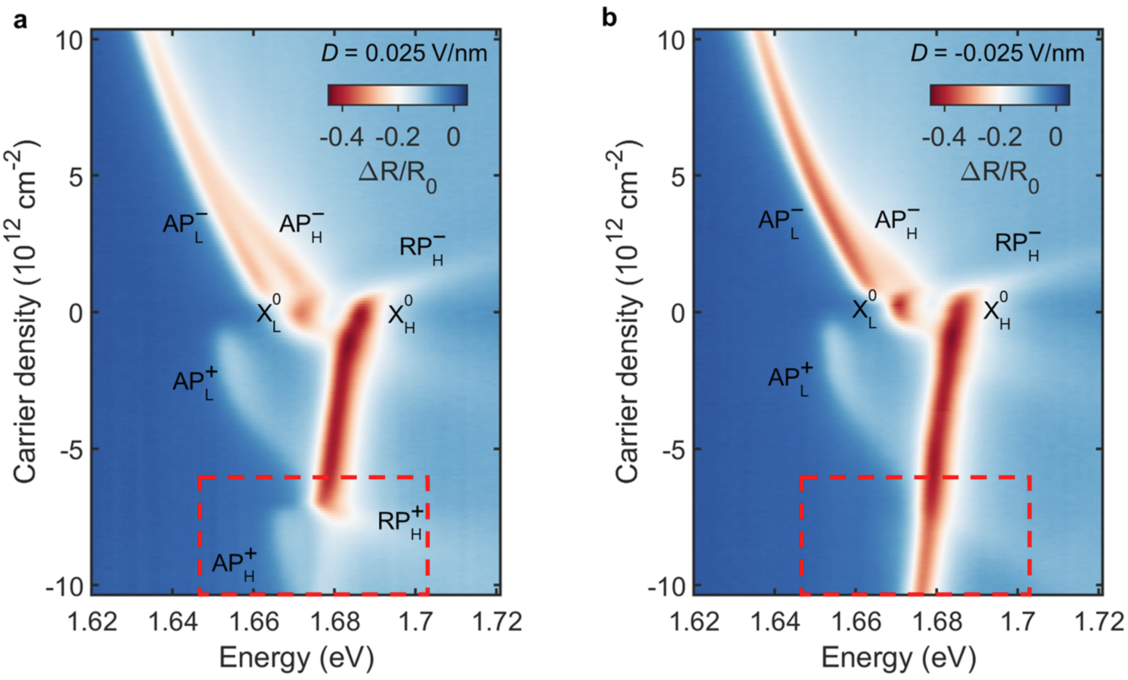

Supplementary Figure 8: Doping dependence of reflectance contrast in a single-domain region under small displacement fields. Reflectance-contrast maps recorded in a single-domain area showing the evolution of excitonic and polaronic resonances with carrier density. **a**, Doping dependence measured under a positive displacement field ($D > 0$). **b**, Doping dependence measured under a negative displacement field ($D < 0$). These measurements demonstrate asymmetry in carrier filling and spectral response when tuning the interlayer potential in a single-domain region. The areas that changed with Fig. 3a are marked by the red dashed boxes.

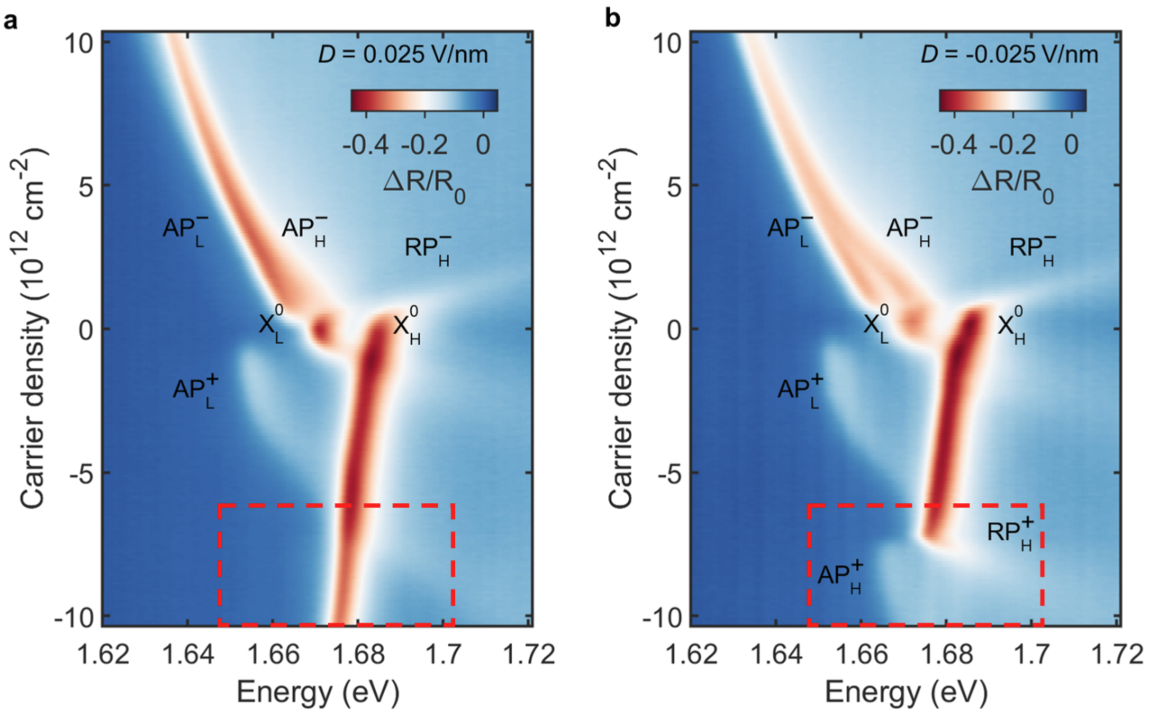

Supplementary Figure 9: Doping dependence after domain switch by a training field (0.15 $\mathrm{V\,nm^{-1}}$). **a**, Doping dependence under a small positive displacement field ($D > 0$) after the training procedure. **b**, Doping dependence under a small negative displacement field ($D < 0$) after the training procedure. Comparison with Fig. 8 highlights spectral changes associated with ferroelectric domain switch. The areas that changed with Fig. 3a are marked by the red dashed boxes.

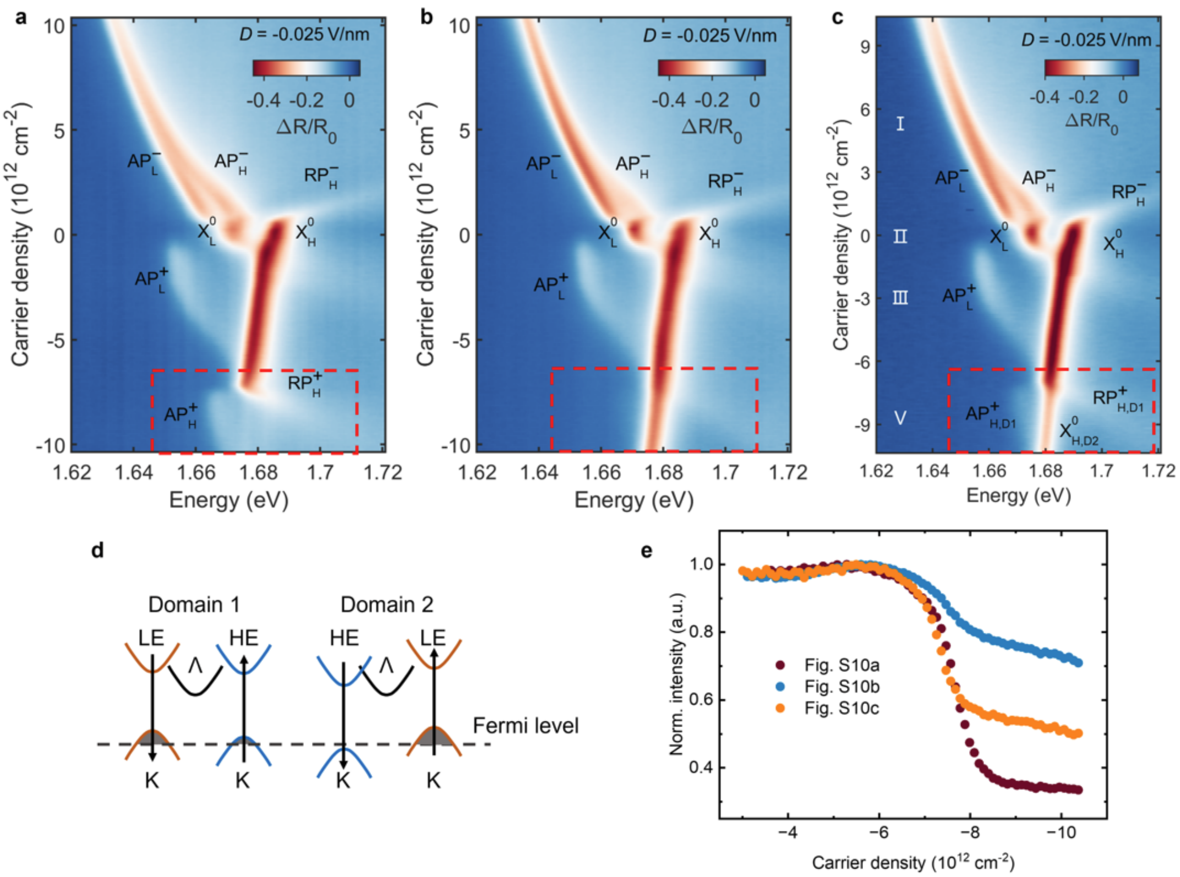

Supplementary Figure 10: Direct comparison of multi-domain and single-domain responses at a fixed electric field. Doping dependent reflectance spectra from (**a**) single domain Type 1, (**b**) single domain Type 2 and (**c**) multi-domain region. The red dashed boxes highlight the high hole-doping regime. The simultaneous observation of two opposite responses in (**c**) serves as definitive proof that this area contains both AB and BA domains. (**d**) Schematic showing the opposite response of AB and BA domains to an external electric field. (**e**) Normalized peak intensity of the high-energy neutral exciton, plotted as a function of doping concentration.

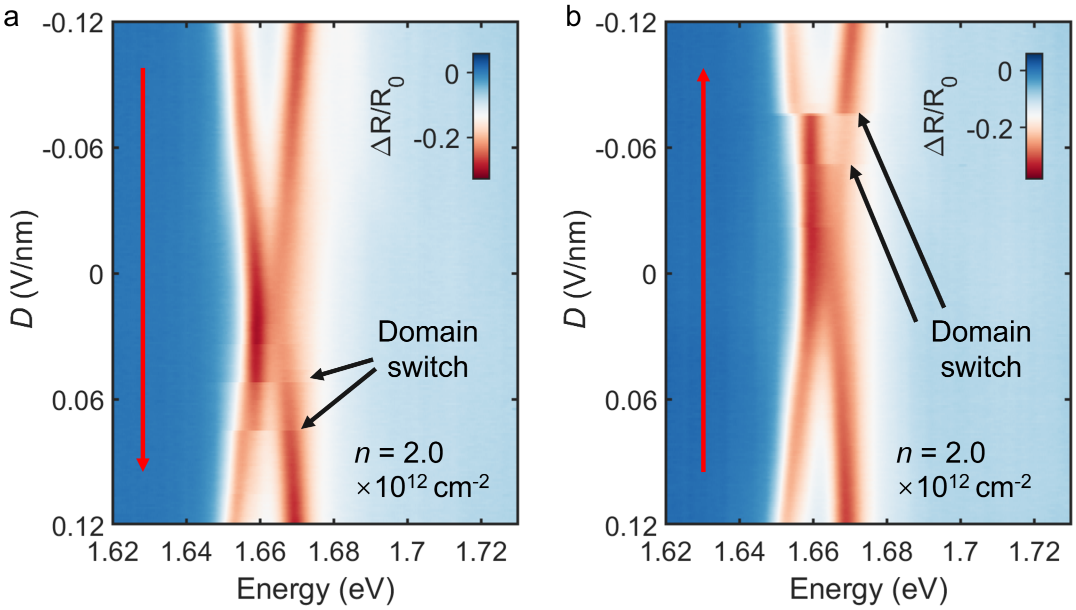

Supplementary Figure 11: Ferroelectric switching in n-doped R-stacked bilayer WSe_2. (**a**, **b**) Reflectance spectrum as a function of the displacement field D. The energies of the attractive polarons (AP) are tracked. (**a**) The forward sweep of the D field, from -0.12 V/nm to +0.12 V/nm. (**b**) The backward sweep of the D field, from +0.12 V/nm to -0.12 V/nm. The sweep direction is indicated by the red arrows. In both panels, two abrupt, discontinuous jumps in the polaron energies are observed, which are the characteristic signatures of ferroelectric domain switching. The presence of two jumps in each direction suggests a multi-step process for the domain wall to traverse the optical spot.

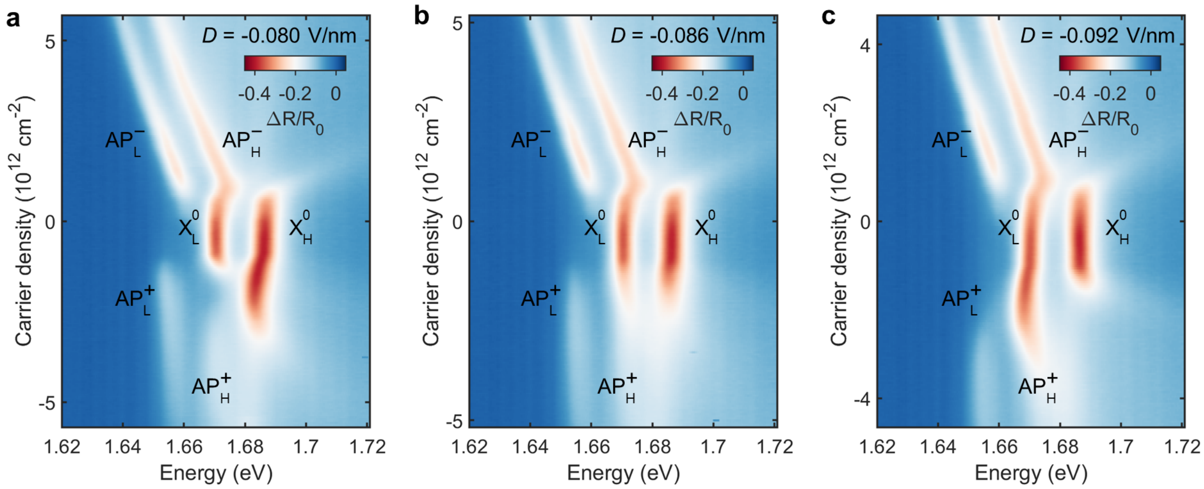

Supplementary Figure 12: Doping dependence near the valence-band-maximum switching field. Doping dependent reflectance contrast maps recorded at three displacement fields near the valence-band maximum (VBM) switching threshold. **a**, $D = -0.080\ \mathrm{V\,nm^{-1}}$. **b**, $D = -0.086\ \mathrm{V\,nm^{-1}}$. **c**, $D = -0.092\ \mathrm{V\,nm^{-1}}$. At $D = -0.080\ \mathrm{V\,nm^{-1}}$ holes preferentially populate the low-energy layer; at $D = -0.092\ \mathrm{V\,nm^{-1}}$ holes preferentially populate the high-energy layer. At the intermediate field $D = -0.086\ \mathrm{V\,nm^{-1}}$ both layers are populated concurrently, indicating the crossover where the valence-band maximum switches layer character.

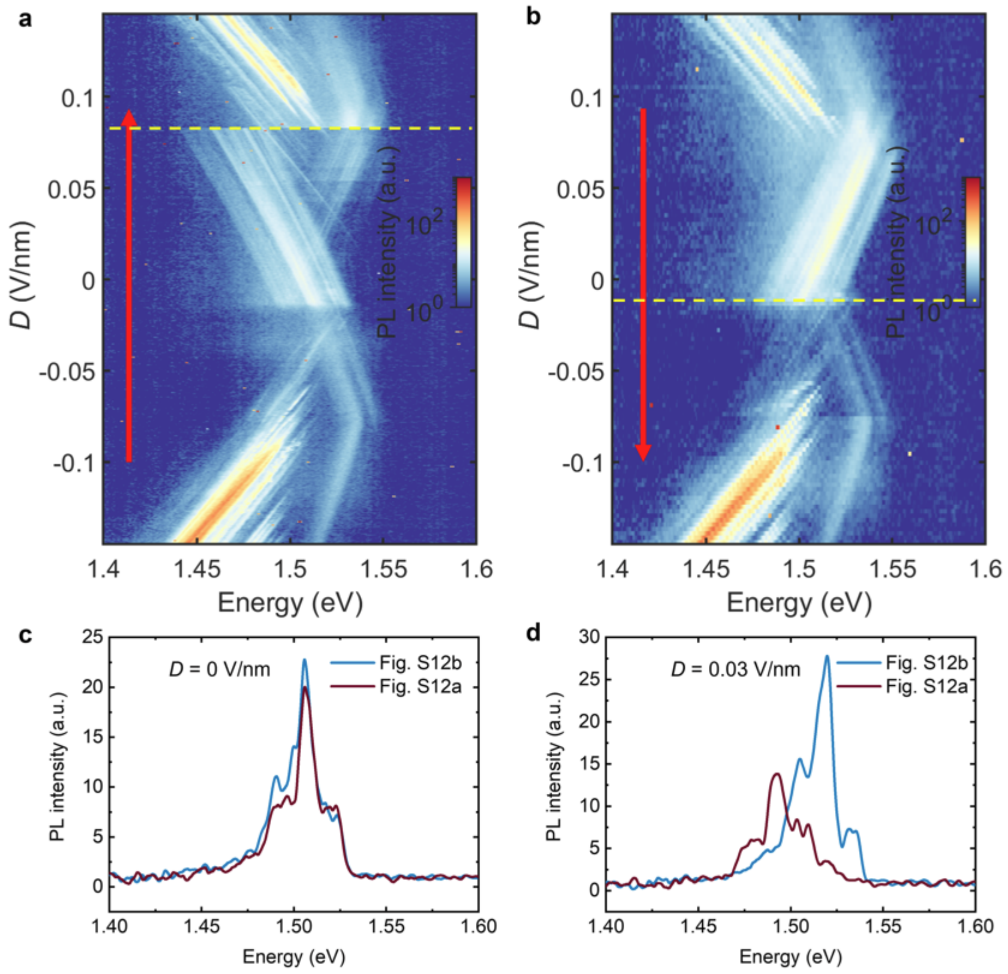

Supplementary Figure 13: Displacement field dependent interlayer exciton emission. Interlayer PL spectrum with scanning D field forward (**a**) and backward (**b**). The scan directions are indicated by red arrows. Extracted spectra under 0 V/nm (**c**) and 0.03 V/nm (**d**) displacement field. A discontinuity in the IX emission energy, highlighted by the dashed yellow line, signals ferroelectric domain switching. Furthermore, this field-dependent measurement provides an unambiguous method to identify the physical location of the LE and HE layers. The IX consists of an electron in the Λ valley (spatially located between the layers) and a hole localized in the K valley of the LE layer. A redshift (blueshift) under positive D indicates the hole is in the bottom (top) layer, meaning the bottom (top) layer is the LE layer.

* Electronic address: B.D.Gerardot@hw.ac.uk
